# Lactate Clearance and Vasopressor Seem to Be Predictors for Mortality in Severe Sepsis Patients with Lactic Acidosis Supplementing Sodium Bicarbonate: A Retrospective Analysis

**DOI:** 10.1371/journal.pone.0145181

**Published:** 2015-12-21

**Authors:** Su Mi Lee, Seong Eun Kim, Eun Bin Kim, Hyo Jin Jeong, Young Ki Son, Won Suk An

**Affiliations:** 1 Department of Internal Medicine, Dong-A University, Busan, Korea; 2 Institute of Medical Science, Dong-A University College of Medicine, Busan, Korea; Azienda Ospedaliero-Universitaria Careggi, ITALY

## Abstract

**Introduction:**

Initial lactate level, lactate clearance, C-reactive protein, and procalcitonin in critically ill patients with sepsis are associated with hospital mortality. However, no study has yet discovered which factor is most important for mortality in severe sepsis patients with lactic acidosis. We sought to clarify this issue in patients with lactic acidosis who were supplementing with sodium bicarbonate.

**Materials and Methods:**

Data were collected from a single center between May 2011 and April 2014. One hundred nine patients with severe sepsis and lactic acidosis who were supplementing with sodium bicarbonate were included.

**Results:**

The 7-day mortality rate was 71.6%. The survivors had higher albumin levels and lower SOFA, APACHE II scores, vasopressor use, and follow-up lactate levels at an elapsed time after their initial lactate levels were checked. In particular, a decrement in lactate clearance of at least 10% for the first 6 hours, 24 hours, and 48 hours of treatment was more dominant among survivors than non-survivors. Although the patients who were treated with broad-spectrum antibiotics showed higher illness severity than those who received conventional antibiotics, there was no significant mortality difference. 6-hour, 24-hour, and 48-hour lactate clearance (HR: 4.000, 95% CI: 1.309–12.219, *P* = 0.015) and vasopressor use (HR: 4.156, 95% CI: 1.461–11.824, *P* = 0.008) were significantly associated with mortality after adjusting for confounding variables.

**Conclusions:**

Lactate clearance at a discrete time point seems to be a more reliable prognostic index than initial lactate value in severe sepsis patients with lactic acidosis who were supplementing with sodium bicarbonate. Careful consideration of vasopressor use and the initial application of broad-spectrum antibiotics within the first 48 hours may be helpful for improving survival, and further study is warranted.

## Introduction

Sepsis is the most common cause of lactic acidosis, and septic patients with lactic acidosis show a higher mortality rate [1, 2]. The etiology of lactic acidosis in sepsis is complex. It may result from either impaired lactate clearance or increased lactate production [3]. Therefore, increased or sustained lactate levels represent severe sepsis or septic shock. In addition, several laboratory tests may be used to assess the sepsis severity or prognosis.

Leukocytosis, elevated C-reactive protein (CRP), and increased procalcitonin are known as traditional markers for sepsis [4, 5]. Lactate level has also been used as a prognostic indicator for mortality [6–9]. In particular, the patients with an initial lactate level > 4.0 mmol/L had higher mortality risks, and the probability of death was substantially increased with a high initial lactate level [8]. Some of the studies reported that lactate clearance, derived from calculating the change in lactate levels at different times, may has potential prognostic value [1, 10]. These studies have proved that a decrease in these markers within the first several hours may be predictive of a favorable outcome. However, no study has yet examined which factor is the most important mortality risk factor among initial lactate, lactate clearance, or inflammatory markers in severe sepsis patients with lactic acidosis.

The use of sodium bicarbonate as a corrector for lactic acidosis remains controversial [11] because sodium bicarbonate may increase lactate production. Previous study has shown that the administration of sodium bicarbonate may negatively affect survival [12]. Lactic acidosis patients supplementing with sodium bicarbonate tend to have more critical and severe conditions than patients who do not receive sodium bicarbonate administration. There are a few data about mortality prognostic factors in patients with lactic acidosis who receive sodium bicarbonate supplementation [12]. Furthermore, it is not clear whether initial lactate level or change in lactate clearance has an effect on mortality in critically ill patients supplementing with sodium bicarbonate because of lactic acidosis.

To clarify the relevance of initial lactate levels, change of lactate levels, and inflammatory markers for mortality, we undertook this retrospective study in severe sepsis patients with lactic acidosis who receive supplementation with sodium bicarbonate. In addition, we evaluated whether vasopressor use, different types of antibiotics, or culture organisms are related to the clinical courses of severe sepsis patients with lactic acidosis.

## Materials and Methods

### Patient Inclusion and Data Collection

We screened 230 patients who had been diagnosed with lactic acidosis, who were being treated with sodium bicarbonate, and who were over 18 years of age between May 2011 and April 2014 at Dong-A University Hospital, Busan, Korea. We defined lactic acidosis as a lactate level >30 mg/dL (3.3 mmol/L) with high anion gap metabolic acidosis. In our hospital laboratory, a high normal lactate level is considered to be 19.8 mg/dL (2.2 mmol/L); therefore, we selected septic patients who had a lactate level >3.3 mmol/L to exclude patients with equivocally high lactate levels. The criteria for exclusion from this study were patients with hyperlactatemia without high anion gap metabolic acidosis. Sepsis was defined as a suspected infection in the presence of two or more systemic inflammatory response syndrome criteria [13]. Finally, 109 patients were included in our analysis.

We retrospectively analyzed the patients’ medical records, including information about the patients’ underlying diseases, laboratory findings, sodium bicarbonate administration, vasopressor and antibiotic use, ventilator care, continuous renal replacement therapy (CRRT), cause of sepsis and pathologic organisms, presence of death, and time to death. CRRT was generally delivered via continuous veno-venous haemodiafiltration using Prismaflex ST100 filters (Gambro Lundia AB, Lund, Sweden) with Hemosol^−^ (Gambro Undia AB). We checked the patients’ age, sex, and vital signs, which included measurements of mean arterial pressure, heart rate, blood temperature, and respiratory rate (RR) at the time of the lactic acidosis diagnosis. We specifically analyzed hemoglobin (Hb), albumin, liver function tests, CRP, procalcitonin, blood urea nitrogen (BUN), and creatinine (Cr). We defined the primary end point as 7-day mortality because most patients with lactic acidosis die soon after diagnosis. Mortality information was obtained from hospital records. Patients who died within 7 days were regarded as non-survivors. Secondary outcome was defined as the relevance of vasopressor use, different types of antibiotics, or culture organisms for mortality. This study was approved by the Dong-A University Institutional Review Board. Informed consent was waived because the study is of a retrospective design. The data including patient records and information was anonymized and de-identified prior to analysis. All clinical investigations were performed in accordance with the Declaration of Helsinki.

### Analysis for Disease Severity and Status of Acidosis

We used SOFA (Sequential Organ Failure Assessment) and APACHE (Acute Physiology And Chronic Health Evaluations)-II scores to estimate illness severity [14, 15]. Therefore, we analyzed initial and follow-up arterial blood gas (PO_2_, PCO_2_, pH, bicarbonate), hematocrit, and white blood cell and platelet counts. We divided vasopressor use up into dopamine, norepinephrine, vasopressin, or phenylephrine use within 12 hours after lactic acidosis diagnosis. We also evaluated using a vasopressor at 24 hours and 48 hours after the diagnosis of lactic acidosis.

### Lactate Level and Lactate Clearance

We were able to check lactate levels at our hospital starting in May 2011, so we could promptly diagnose lactic acidosis after that time. Lactate levels were measured using Roche/Hitachi912/MODULAR P analyzers (CAN 040, Roche, Indianapolis, USA). We measured serial lactate levels at 6, 24, and 48 hours after checking the initial lactate level. Lactate clearance was calculated by the equation: [(lactate_initial_-lactate_follow-up_)/lactate_initial_] *100%. Lactate_initial_ was defined as the measurement at the time of lactic acidosis diagnosis, and Lactate_follow-up_ was the measurement at 6, 24, and 48 hours after lactate_initial_. We evaluated lactate clearance as being a deficit of at least 10% [1, 10].

### Antibiotics and Culture Organisms Group

We stratified the patients into 2 groups depending on whether they received broad-spectrum antibiotics treatment—including vancomycin, teicoplanin, or carbapenem—at the time of lactic acidosis diagnosis or changed their treatment within 48 hours as part of their early goal-directed therapy. Conventional antibiotic treatment included use of beta-lactam antibiotics, quinolones, metronidazole, or azotreonam. In addition, we identified the characteristic differences between the patients who were positive and negative for etiologic organism by culture.

### Statistical Analysis

Data were expressed as mean ± SD or frequency. The subjects’ characteristics were analyzed using Student’s *t* test for continuous variables. A Chi-squared test was used to compare categorical data between the 2 groups. Receiver operating characteristic (ROC) analysis by Youden Index was used to explore the diagnostic performance of lactate levels for the determination of mortality [16]. To identify cumulative mortality risk according to lactate levels, Kaplan-Meier analyses and log rank tests were performed. Furthermore, a multivariate Cox proportional hazards regression analysis was applied to identify the association between lactate clearance and mortality. The variables included age, sex, CRP, albumin, SOFA and APACHE II scores, ventilator care, CRRT, vasopressor use, and lactate clearance. We separately analyzed factors associated with mortality using independent models of Cox proportional analysis based on lactate clearance at discrete time points.

The generalized estimating equation (GEE) model was fitted to predict the mortality with regard to the clearance of lactate [17]. Lactate clearance were transformed to binary data by converting values >10% or <10% to 0 or 1. This statistical model uses a repeated-measures design to account to correlated observations. Adjustment covariates for these model was age, sex, CRP, albumin, SOFA and APACHE II scores, ventilator care, and CRRT. A *P* value < 0.05 was considered to be statistically significant. All statistical calculations were performed with SPSS software (SPSS version 18.0, Chicago, IL).

## Results

### Baseline Characteristics

Among the 230 patients with lactic acidosis who were supplementing with sodium bicarbonate, we finally diagnosed 109 (47.4%) with severe sepsis during the 3-year investigational period from 2011 to 2014. Baseline characteristics are presented in Table 1. Of all the enrolled patients, 94 (86.2%) died, and the median survival time was 2 days (1–57 days) among the non-survivors (data not shown). The mean patient age was 64.4 ± 14.2 years, and 71.6% of the study population was male. The causes of sepsis were lung (36.7%), gastrointestinal (29.4%), urinary tract (6.4%), skin and soft tissue (9.2%), catheter-related infection (8.3%), and others (7.3%). Of the enrolled patients, 33.9% had diabetes mellitus, 10.1% had heart failure, 11.9% had liver cirrhosis, and 5.5% had end-stage renal disease (ESRD). The percentage of patients with at least two comorbidities was 13.7%. The initial pH was 7.23 ± 0.17, bicarbonate was 13.7 ± 5.6 mEq/L, PCO_2_ was 33.9 ± 15.7 mm Hg, lactate level was 78.3 ± 39.6 mg/dL, and the anion gap was 20.7 ± 13.4. The average SOFA score was 12.0 ± 3.9, and the average APACHE II score was 29.9 ± 7.8. A vasopressor was used with 91 patients (83.5%). Seventy-six (69.7%) patients received mechanical ventilation, and 34 (32.1%) were treated with CRRT.

**Table 1:** Baseline characteristics.

	Total
	(n = 109)
Age (years)	64.4±14.2
Male, n (%)	78 (71.6)
Diabetes mellitus, n (%)	37 (33.9)
Heart failure, n (%)	11 (10.1)
Liver cirrhosis, n (%)	13 (11.9)
End-stage renal disease, n (%)	6 (5.5)
Cause of sepsis, n (%)	
Lung	40 (36.7)
Gastrointestinal	32 (29.4)
Urinary tract	7 (6.4)
Skin and soft tissue	10 (9.2)
Catheter/blood stream	9 (8.3)
Others	8 (7.3)
Initial pH	7.23±0.17
Initial bicarbonate (mEq/L)	13.7±5.6
Initial PCO_2_ (mm Hg)	33.9±15.7
Initial lactate (mg/dL)	78.3±39.6
Anion gap	20.7±13.4
Albumin (g/dL)	2.9±0.7
Creatinine (mg/dL)	2.8±2.0
Glomerular filtration rate (mL/min/1.73m^2^)	37.1±29.1
C-reactive protein (mg/dL)	17.0±12.5
Procalcitonin (ng/mL)	33.8±52.2
Ejection fraction (%)	54.5±13.9
SOFA	12.0±3.9
APACHE II	29.9±7.8
Ventilator care, n (%)	76 (69.7)
CRRT, n (%)	34 (32.1)
Vasopressor use, n (%)	91 (83.5)
Antibiotics (Vancomycin/Teicoplanin/Carbapenem), n (%)	53 (48.6)
Culture positive, n (%)	66 (59.6)
Total dose of sodium bicarbonate (mEq)	215.3±248.1

SOFA, Sequential Organ Failure Assessment; APACHE, Acute Physiology and Chronic Health Evaluation; CRRT, continuous renal replacement therapy

### Primary Outcome

**Fig 1:**
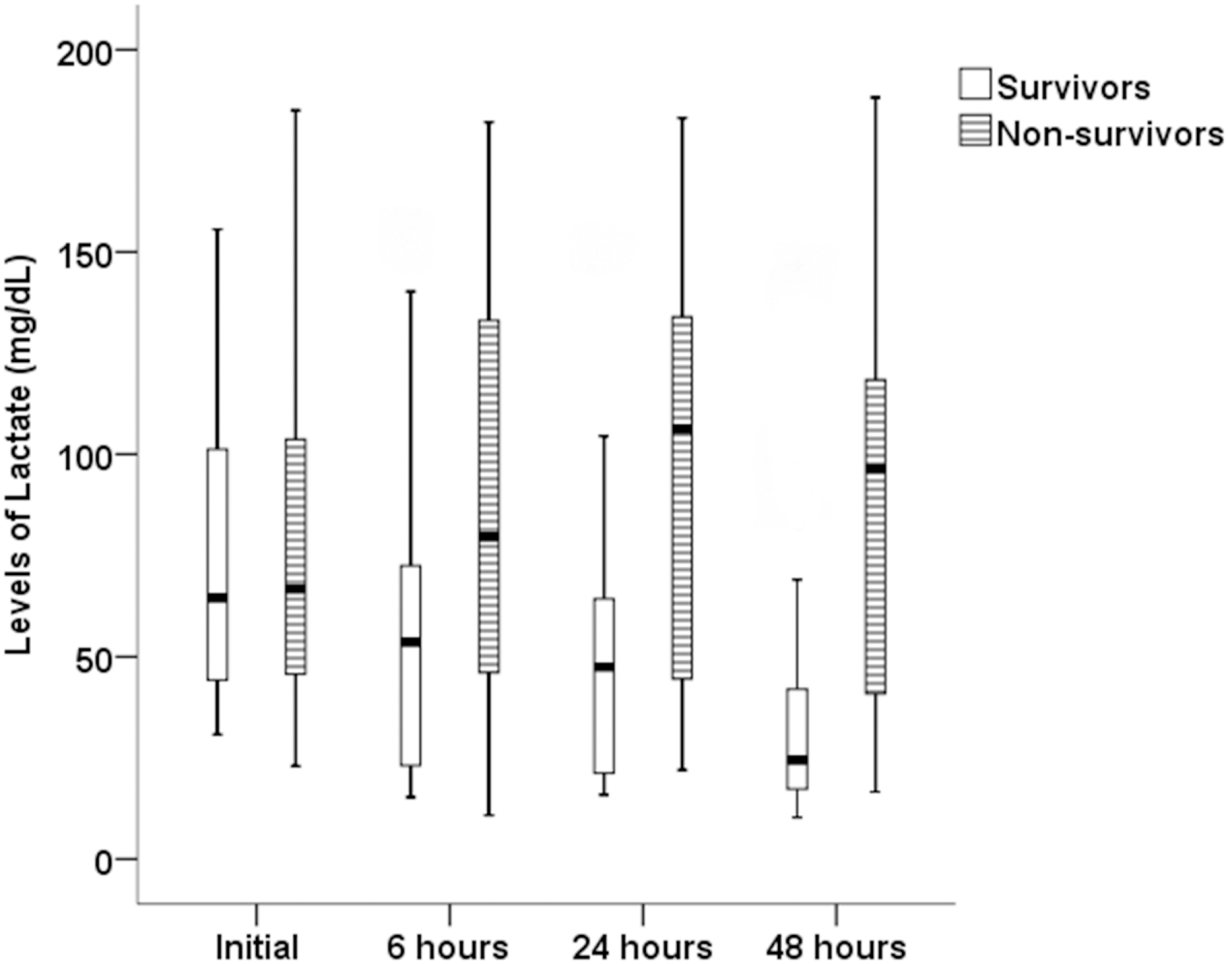
Changes of lactate levels over time.

The 7-day mortality rate was 71.6%, and the follow-up lactate level was 78.6 ± 49.0 mg/dL at 6 hours, 78.1 ± 51.1 mg/dL at 24 hours, and 63.2 ± 50.4 mg/dL at 48 hours. As detailed in Table 2, statistically significant differences were apparent in the clinical markers between survivors and non-survivors. The non-survivors had lower albumin levels (*P* = 0.009), higher SOFA and APACHE II scores (*P* = 0.002, *P* = 0.047, respectively), higher vasopressor use (*P* < 0.001), and higher lactate levels at 6, 24, and 48 hours after the initial lactate level was checked (*P* = 0.002, *P* < 0.001, *P* = 0.001, respectively). In particular, a decrement of at least 10% in lactate clearance for the first 6, 24, and 48 hours after treatment was more prominent in the survival group than the non-survival group (Table 2). There were no significant differences in initial pH, initial bicarbonate, initial PCO_2_, CRP, procalcitonin, initial and maximum lactate levels, causes of sepsis, and use of mechanical ventilator and CRRT between the survivors and non-survivors. Survivors’ lactate levels significantly decreased over time (*P* = 0.002); however, this was not the case for the non-survivors (*P* = 0.357; Fig 1).

**Table 2:** Patient characteristics according to 7-day mortality.

	Survivors	Non-survivors	*P* Value^a^
	(n = 29)	(n = 78)	
Age (years)	65.7±14.4	64.8±14.1	0.526
Male, n (%)	21 (72.4)	56 (71.8)	0.949
Diabetes mellitus, n (%)	11 (37.9)	25 (32.1)	0.567
Heart failure, n (%)	2 (6.9)	9 (11.5)	0.482
Liver cirrhosis, n (%)	3 (10.3)	10 (12.8)	0.728
End-stage renal disease, n (%)	2 (6.9)	4 (5.1)	0.724
Cause of sepsis, n (%)			0.622
Lung	12 (41.4)	27 (34.6)	0.518
Gastrointestinal	8 (27.6)	24 (30.8)	0.749
Urinary tract	1 (3.4)	6 (7.7)	0.430
Skin and soft tissue	4 (13.8)	6 (7.7)	0.335
Catheter/blood stream	1 (3.4)	7 (9.0)	0.334
Others	2 (6.9)	6 (7.7)	0.889
Initial pH	7.28±0.17	7.21±0.16	0.071
After 48 hours pH	7.39±0.24	7.17±0.21	0.006
Initial bicarbonate (mEq/L)	13.7±5.0	13.7±5.9	0.992
After 48 hours bicarbonate (mEq/L)	20.0±7.3	17.1±7.6	0.239
Initial PCO_2_ (mm Hg)	32.3±15.8	34.8±15.8	0.471
After 48 hours PCO_2_ (mm Hg)	31.1±10.9	44.6±22.8	0.041
Initial lactate (mg/dL)	77.0±39.4	78.8±39.6	0.833
After 6 hours lactate (mg/dL)	57.1±38.0	87.8±50.9	0.002
After 24 hours lactate (mg/dL)	50.1±34.9	95.2±52.4	<0.001
After 48 hours lactate (mg/dL)	38.6±33.8	86.8±52.7	0.001
Max lactate (mg/dL)	88.9±41.6	106.6±43.9	0.063
Lactate clearance at 6 hours <10%	9 (32.1)	43 (68.3)	0.001
Lactate clearance at 24 hours <10%	7 (29.2)	27 (75.0)	<0.001
Lactate clearance at 48hours <10%	4 (16.0)	15 (65.2)	<0.001
Anion gap	20.6±5.9	20.7±15.5	0.962
Albumin (g/dL)	3.2±0.6	2.8±0.7	0.009
Creatinine (mg/dL)	2.6±1.6	2.9±2.2	0.465
Glomerular filtration rate (mL/min/1.73m^2^)	40.0±35.4	35.5±26.2	0.501
C-reactive protein (mg/dL)	17.7±10.6	16.6±13.4	0.689
Procalcitonin (ng/mL)	45.2±57.5	30.0±50.9	0.263
SOFA	10.2±3.5	12.7±3.8	0.002
APACHE II	27.6±8.3	31.0±7.5	0.047
Ventilator care, n (%)	19 (65.5)	56 (71.8)	0.528
CRRT, n (%)	8 (27.6)	26 (33.3)	0.570
Vasopressor use, n (%)	18 (62.1)	71 (91.0)	<0.001
Sustained use of vasopressor after 24 hours	7 (24.1)	65 (83.3)	<0.001
Sustained use of vasopressor after 48 hours	3 (10.3)	33 (80.5)	<0.001
Antibiotics (Vancomycin/Teicoplanin/Carbapenem), n (%)	23 (79.3)	64 (82.1)	0.746
Anti-fungal agent, n (%)	3 (10.3)	8 (10.3)	0.989
Culture positive, n (%)	18 (62.1)	46 (59.0)	0.772
Total dose of sodium bicarbonate (mEq)	213.1±250.8	216.6±250.8	0.949

SOFA, Sequential Organ Failure Assessment; APACHE, Acute Physiology and Chronic Health Evaluation; CRRT, continuous renal replacement therapy

^*a*^Comparison between survivors and nonsurvivors.

**Fig 2:**
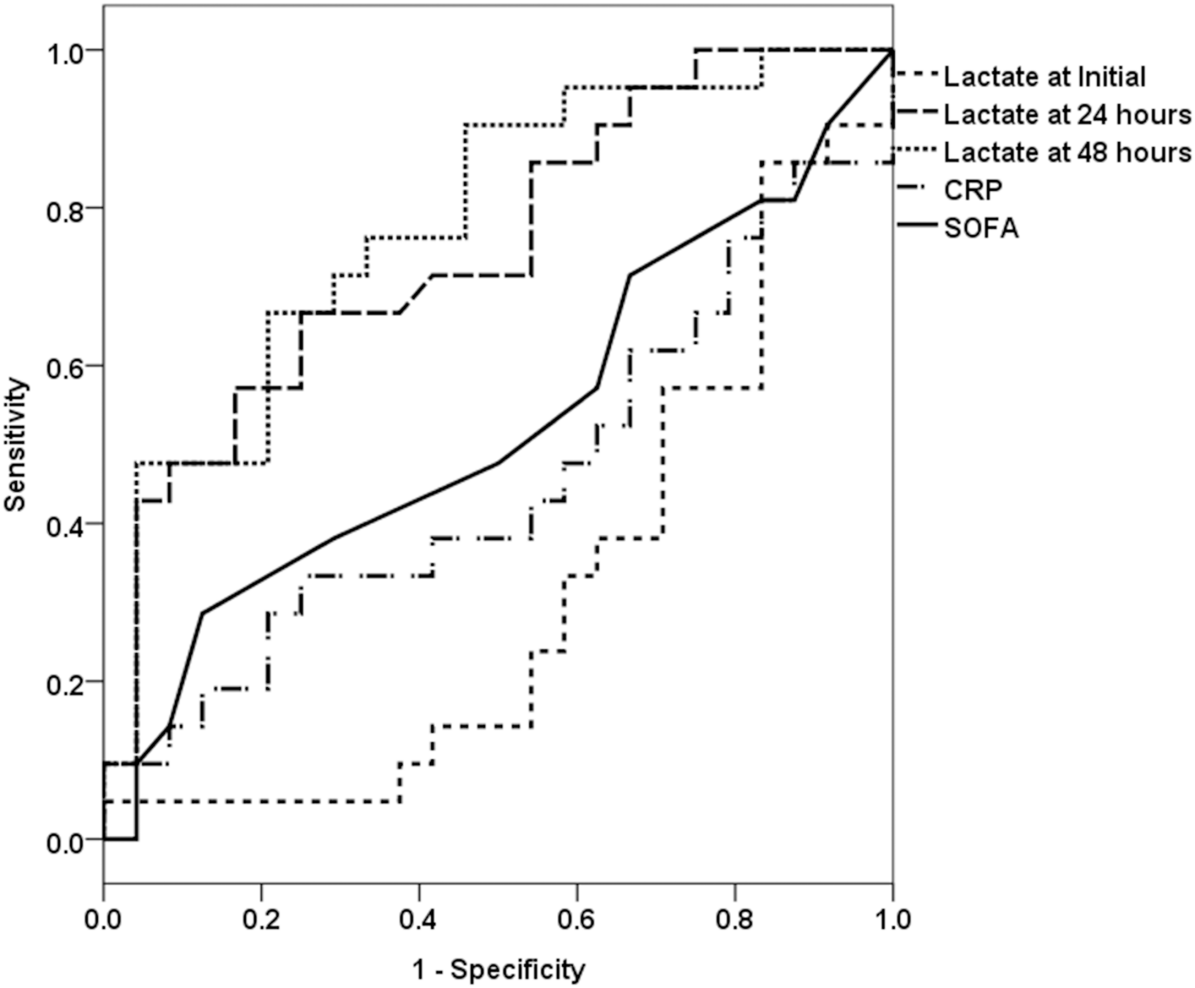
Receiver operating characteristic curves for lactate levels predicting 7-day mortality.

We compared the diagnostic performance of lactate or follow-up lactate levels, CRP, and SOFA scores for the prediction of 7-day mortality through ROC analysis (Fig 2). Lactate levels at 24 and 48 hours showed higher area under the ROC curves (AUC) than did the initial lactate level, CRP, and SOFA scores. The AUCs for lactate levels at 24 and 48 hours to predict 7-day mortality were 0.749 (95% confidence interval [95% CI]: 0.606–0.892, *P* = 0.004) and 0.782 (95% CI: 0.647–0.917, *P* = 0.001). In addition, best Youden index for 7-day mortality were lactate levels at 24 and 48 hours (0.458 and 0.496).

### Patient Characteristics According to Antibiotics

Patients were categorized as being in either the broad-spectrum (vancomycin, teicoplanin, or carbapenem) or conventional antibiotics group (Table 3). The patients treated with broad-spectrum antibiotics had higher tendency toward initial CRP and procalcitonin (*P* = 0.085, *P* < 0.001, respectively) and follow-up CRP and procalcitonin (*P* = 0.009, *P* = 0.309, respectively), higher SOFA scores (*P* = 0.021), and a greater tendency toward vasopressor use (*P* = 0.072). However, 7-day mortality rates were not significantly different between the two groups. The impact of antibiotics on 7-day mortality was evaluated using a Kaplan-Meier analysis. There was no significant association between kinds of antibiotics and mortality (*P* = 0.958, log-rank test).

**Table 3:** Patient characteristics according to antibiotics.

	conventional antibiotics	broad-spectrum antibiotics	*P* Value^a^
	(n = 20)	(n = 89)	
Age (years)	64.6±16.0	64.3±13.8	0.947
Male, n (%)	15 (75.0)	63 (70.8)	0.706
Diabetes mellitus, n (%)	4 (20.0)	33 (37.1)	0.145
Heart failure, n (%)	4 (20.0)	7 (7.9)	0.104
Liver cirrhosis, n (%)	2 (10.0)	11 (12.4)	0.769
End-stage renal disease, n (%)	0 (0)	6 (6.7)	0.232
Cause of sepsis, n (%)			0.394
Lung	8 (40.0)	32 (36.0)	0.735
Gastrointestinal	7 (35.0)	25 (28.1)	0.540
Urinary tract	2 (10.0)	5 (5.6)	0.470
Skin and soft tissue	1 (5.0)	9 (10.1)	0.474
Catheter/blood stream	1 (5.0)	8 (9.0)	0.558
Others	1 (5.0)	7 (7.9)	0.657
Initial pH	7.24±0.14	7.23±0.17	0.790
Initial bicarbonate (mEq/L)	15.2±7.8	13.4±5.0	0.325
Initial PCO_2_ (mm Hg)	35.5±16.7	33.5±15.6	0.614
Initial lactate (mg/dL)	78.4±34.9	78.3±40.7	0.996
After 6 hours lactate (mg/dL)	76.7±59.1	79.0±47.2	0.869
After 24 hours lactate (mg/dL)	74.6±60.9	78.6±50.1	0.838
After 48 hours lactate (mg/dL)	63.2±65.1	63.2±48.5	1.000
Max lactate (mg/dL)	103.5±46.9	102.1±43.1	0.902
Lactate clearance at 6 hours <10%	8 (53.3)	45 (58.4)	0.714
Lactate clearance at 24 hours <10%	3 (37.5)	32 (60.4)	0.223
Lactate clearance at 48hours <10%	2 (28.6)	18 (42.9)	0.476
Anion gap	20.1±7.7	20.8±14.5	0.836
Albumin (g/dL)	2.9±0.9	2.9±0.6	0.957
Creatinine (mg/dL)	2.1±0.8	3.0±2.2	0.003
Glomerular filtration rate (mL/min/1.73m^2^)	39.8±21.8	36.4±30.6	0.642
C-reactive protein (mg/dL)	12.4±0.9	18.0±12.6	0.085
C-reactive protein (mg/dL) f/u	8.0±5.5	15.9±10.4	0.009
Procalcitonin (ng/mL)	4.7±5.9	39.5±55.2	<0.001
Procalcinonin f/u	13.5±22.9	39.8±49.0	0.309
SOFA	10.2±3.4	12.4±3.9	0.021
APACHE II	27.8±8.4	30.4±7.7	0.175
Ventilator care, n (%)	16 (80.0)	60 (67.4)	0.268
CRRT, n (%)	5 (25.0)	30 (33.7)	0.451
Vasopressor use, n (%)	14 (70.0)	77 (86.5)	0.072
7 days mortality, n(%)	14 (70.0)	64 (73.6)	0.746
Culture positive, n (%)	8 (40.0)	58 (65.2)	0.037
Anti-fungal agent, n (%)	0 (0)	11 (12.4)	0.097
Total dose of sodium bicarbonate (mEq)	214.7±239.9	215.4±251.3	0.991

f/u, followed up within 72 hours; SOFA, Sequential Organ Failure Assessment; APACHE, Acute Physiology and Chronic Health Evaluation; CRRT, continuous renal replacement therapy

^*a*^Comparison between conventional antibiotics and broad-spectrum antibiotics.

### Patient Characteristics According to Culture Organism

Of all the enrolled patients, 66 (60.6%) had culture positive infections (data not shown). Among the patients with culture positive infection, the patients with multi-resistant germs were 39 (59.1%) and the patients treated with adequate antibiotics use was 55 (83.3%). Analyzing the relationship between antibiotic type and adequate antibiotic use or percentage of multi-resistant germs, broad-spectrum antibiotics group had higher proportion of adequate antibiotic use than conventional antibiotics group (87.9% vs. 50.0%, P = 0.007). In terms of multi-resistant germs, there was a no significant difference between two groups. Forty-three (39.4%) showed culture negative infections. Culture negative infections were found in the lung (48.8%), gastrointestinal (30.2%), urinary tract (2.3%), skin and soft tissue (4.7%), and other areas (14.0%). The lungs were the dominant foci of sepsis caused by culture negative organisms, compared with culture positive organisms (*P* = 0.034), while catheter-related infections were only present among the culture positive infections (*P* = 0.011). There were no significant differences in demographic factors, lactate levels, SOFA and APACHE II scores, or 7-day mortality rates between the two groups.

### Prediction of Mortality by Lactate Levels: Multivariate Models

The independent effect of lactate levels on mortality was examined using multivariate Cox proportional hazard models (Table 4). The lactate clearance at a discrete time point remained as an independent variable associated with mortality after adjusting for confounding variables, including age, sex, CRP, albumin, SOFA and APACHE II scores, ventilator care, vasopressor use, and CRRT. Lactate clearance at 6, 24, and 48 hours were significantly associated with mortality (hazard ratio [HR]: 2.390, 95% CI: 1.296–4.405, *P* = 0.005; HR: 3.206, 95% CI: 1.146–8.968, *P* = 0.026; and HR: 4.000, 95% CI: 1.309–12.219, *P* = 0.015, respectively). Vasopressor use was also significantly associated with mortality (hazard ratio [HR]: 4.156, 95% CI: 1.461–11.824, *P* = 0.008). In terms of lactate clearance using GEE analysis, with the presence of lower lactate clearance, the odds for death was increased relative to higher lactate clearance (odds ratio [OR]:3.052, 95% CI: 1.148–4.956, *P* = 0.002).

**Table 4:** Multivariate Cox proportional model for survival.

	HR^a^ (95% CI)	*P* Value
Age (years)	0.986 (0.966–1.006)	0.164
Male (n/%)	1.405 (0.722–2.733)	0.317
C-reactive protein (mg/dL)	0.997 (0.975–1.019)	0.768
Albumin (g/dL)	0.749 (0.494–1.135)	0.172
SOFA	1.016 (0.911–1.134)	0.769
APACHE II	1.002 (0.951–1.055)	0.955
Ventilator use, n (%)	0.627 (0.298–1.321)	0.220
CRRT, n (%)	0.800 (0.407–1.575)	0.519
Vasopressor use, n (%)	4.156 (1.461–11.824)^b^	0.008
Lactate clearance at 6 hours <10%	2.390 (1.296–4.405)^b^	0.005
Lactate clearance at 24 hours <10%	3.206 (1.146–8.968)^b^	0.026
Lactate clearance at 48hours <10%	4.000 (1.309–12.219)^b^	0.015

SOFA, Sequential Organ Failure Assessment; APACHE, Acute Physiology and Chronic Health Evaluation; CRRT, continuous renal replacement therapy

^a^Clinical parameters (age, gender, c-reactive protein, albumin, SOFA, APACHE II, ventilator use, and CRRT) were examined with lactate clearance.

^b^The effects of variables were examined separately.

## Discussion

In this study, we found that levels of lactate tend to increase, or at least not decrease, with time among non-survivors, but lactate levels significantly decrease over time in survivors after 48 hours. In addition, lactate clearance of at least 10% at 6, 24, and 48 hours and the use of vasopressors are independent factors related to mortality, even after adjusting for critically ill status or sepsis severity. Previous studies also showed that early lactate clearance of equal to or more than 10% at 6 hours is an independent factor related with mortality in septic patients [1, 10]. Our study not only supports these previous findings but also demonstrates the importance of delayed lactate clearance at 24 and 48 hours. Furthermore, we elucidated that lactate clearance was a more important survival-influencing factor than initial or maximum lactate level in critically ill patients supplementing with sodium bicarbonate whose high mean initial lactate level was 8.7 mmol/L. Low lactate clearance reflects the fact that lactate production is more dominant than either lactate excretion or metabolism. Lactate clearance increments during resuscitation may be associated with improved organ functioning, and this suggests a decrease in mortality risk. A lactate clearance of over 10% reflects a tendency to overcome the lactate production caused by a septic condition. Therefore, several strategies are necessary to increase lactate clearance to improve the survival of severe sepsis patients with lactic acidosis that is treated with sodium bicarbonate.

Vasopressors should be avoided in lactic acidosis, if possible, because they may worsen tissue perfusion and increase lactate production. Over sixty percent of survival patients have used vasopressors, but significantly fewer used them compared to the non-survival patients assessed in this study. It is notable that the percentages of survival patients still using vasopressors at 24 and 48 hours were significantly lower compared to those of non-survival patients.

It is well known that higher SOFA and APACHE II scores are related to mortality in critically ill patients. Survival patients in our study definitely had lower SOFA and APACHE II scores than did the non-survival patients. The percentages of patients still using vasopressors and having a lactate clearance less than 10% at 24 and 48 hours had significantly lower survival rates compared to non-survival patients. In addition, initial vasopressor use was another independent factor for 7-day mortality after adjusting for disease severity. These results show that vasopressor use should be decided on only after careful consideration and that making it possible for the patient to gradually taper off vasopressor use may be important for enhancing survival rates, especially among patients with severe sepsis and lactic acidosis who supplement with sodium bicarbonate. Further studies are necessary to more thoroughly examine the role of vasopressors in severe sepsis-related lactic acidosis.

Lactate is a marker for tissue hypoxia. Lactate production results from glycolysis, and it metabolizes by either the liver or the kidney [3, 18]. Lactate production in sepsis increases with glycolysis stimulation or pyruvate metabolism inhibition [19, 20]. During septic shock, glycolytic flux is augmented by epinephrine-dependent stimulation of the beta2-adrenoceptor, and the exaggerated flux is induced by direct enhancement of the NaK-adenosine triphosphatase in the muscle [21]. In addition, lactate clearance is reduced because of multi-organ failure including liver and kidney in sepsis [22]. Previous studies have reported that if patients have an infection, those with lactate levels of ≥4.0 mmol/L have a 38% mortality rate, compared with a mortality rate of 25% for those with lactate levels of 2.0–4.0 mmol/L and 15% for those with lactate levels of ≤2.0 mmol/L [8]. Enrolled patients in our study had higher initial mean lactate levels and mortality rates compared to patients examined in other studies. This may be due to the differences in clinical characteristics of the enrolled patients. The patients were treated with sodium bicarbonate, which is linked to more critical and severe conditions. Initial intermediate and high serum lactate levels were associated with mortality [23]. Although initial lactate level is important, follow-up lactate levels are more important, especially in critically ill patients such as those with lactic acidosis who are supplementing with sodium bicarbonate. In this group, sodium bicarbonate and vasopressor use may additionally increase production of lactate and have an effect on mortality.

Given the potentially deleterious effects of an acidic environment, some clinicians recommend therapy with intravenous sodium bicarbonate for severe acidemia [24]. However, the correction of acidosis by sodium bicarbonate may negatively affect survival and increase lactate production caused by not reducing the enzyme activity of phosphofructokinase in lactic acidosis [25]. The adequate dose and start time of bicarbonate therapy for not increasing lactate production or improving hemodynamics remains controversial [11]. To avoid potential problems, patients supplementing with sodium bicarbonate were enrolled in this study. Our results showed that initial pH, initial bicarbonate, initial PCO_2_, and initial lactate levels were similar between survivors and non-survivors. However, there were significant differences between the two groups in lactate, pH, and PCO_2_ after 48 hours. Decreased pH, no changes in bicarbonate, and increased PCO_2_ may be induced by accompanying respiratory acidosis or incomplete compensatory respiratory alkalosis in non-survival patients. On the contrary, the use of sodium bicarbonate can induce acute hypercapnia if adequate ventilation is not performed. Non-survivors did not show improved acidosis with elapsed time, despite the bicarbonate use in our study. In fact, there were no significant differences in PCO_2 measured_ during the ventilator care between our study’s two groups. Further studies are needed to identify whether a decrement in PCO_2_ using a ventilator can improve acidemia without sodium bicarbonate administration in severe sepsis patients with lactic acidosis.

Recent studies have guided therapy by using changes in lactate levels [26, 27]. Resuscitative efforts should be complemented to treat underlying causes of lactic acidosis [28, 29]. Such efforts include treatment with the appropriate antibiotic agents or intervention. In this study, 5 patients with gastrointestinal infection had percutaneous transhepatic gallbladder drainage, percutaneous transhepatic biliary drainage, endoscopic retrograde cholangiopancreatographic drainage, and pigtail catheter drainage. One patient with urinary tract infection had percutaneous nephrostomy, and one with skin infection had fasciotomy. Of the 7 patients who underwent intervention, 3 died, and the median survival time was 3 days (1–4 days). Four patients survived for a relatively long period of time (median: 398.5 [317–466] days). The early administration of antibiotics in sepsis is strongly recommended by the Surviving Sepsis Campaign [26].

In this study, we identified the ways in which different types of antibiotics may affect clinical courses. The patients were classified into two groups—the broad-spectrum or the conventional antibiotic group. Although conventional antibiotics cover variable-spectrum organisms, broad-spectrum antibiotics including vancomycin, carbapenem, teicoplanin tend to cover a wider range germs. Therefore, authors were defined that vancomycin, carbapenem, and teicoplanin is broad-spectrum antibiotics. The patients treated with broad-spectrum antibiotics had more use of vasopressors and higher CRP and procalcitonin levels, and elevated APACHE II scores, compared to those who were being treated with conventional antibiotics. The broad-spectrum antibiotics group may have had more severe clinical conditions than did the conventional antibiotics group; however, there were no significant differences in mortality between the two groups. This may indicate that the use of broad-spectrum antibiotics is more suitable in initial therapy for severe sepsis and septic shock with lactic acidosis than is the use of conventional antibiotics. To our knowledge, this study is the first to identify the fact that broad-spectrum antibiotic treatment may positively affect outcomes in severe sepsis patients with lactic acidosis. Further prospective studies are needed to confirm these effects.

This study has some limitations because it used a retrospective analysis, and the sample size was relatively small. In addition, interventions in this study were not standardized or controlled. Despite these limitations, we found that measuring serial lactate levels, limiting vasopressor use, and initially using broad-spectrum antibiotics may be useful for severe sepsis patients with lactic acidosis supplementing sodium bicarbonate.

## Conclusions

In conclusion, lactate clearance at a discrete time point seems to be a more reliable prognostic index than an initial lactate value taken alone or disease severity markers, including SOFA and APACHE scores in severe sepsis patients with lactic acidosis who are supplementing with sodium bicarbonate. Careful consideration of vasopressor use and the initial application of broad-spectrum antibiotics within 48 hours may be helpful for improving survival. Further studies are needed concerning critically ill patients with lactic acidosis caused by sepsis.

## References

[1] Nguyen HB, Rivers EP, Knoblich BP, Jacobsen G, Muzzin A, Ressler JA, et al Early lactate clearance is associated with improved outcome in severe sepsis and septic shock. Critical care medicine. 2004;32(8):1637–42. Epub 2004/08/03. . 15286537 10.1097/01.ccm.0000132904.35713.a7

[2] Aduen J, Bernstein WK, Khastgir T, Miller J, Kerzner R, Bhatiani A, et al The use and clinical importance of a substrate-specific electrode for rapid determination of blood lactate concentrations. Jama. 1994;272(21):1678–85. Epub 1994/12/07. . 7966896

[3] Kraut JA, Madias NE. Lactic acidosis. The New England journal of medicine. 2014;371(24):2309–19. Epub 2014/12/11. 10.1056/NEJMra1309483 . 25494270

[4] Mitaka C. Clinical laboratory differentiation of infectious versus non-infectious systemic inflammatory response syndrome. Clinica chimica acta; international journal of clinical chemistry. 2005;351(1–2):17–29. Epub 2004/11/27. 10.1016/j.cccn.2004.08.018 . 15563869

[5] Castelli GP, Pognani C, Meisner M, Stuani A, Bellomi D, Sgarbi L. Procalcitonin and C-reactive protein during systemic inflammatory response syndrome, sepsis and organ dysfunction. Crit Care. 2004;8(4):R234–42. Epub 2004/08/18. 10.1186/cc2877 15312223 PMC522844

[6] Rishu AH, Khan R, Al-Dorzi HM, Tamim HM, Al-Qahtani S, Al-Ghamdi G, et al Even mild hyperlactatemia is associated with increased mortality in critically ill patients. Crit Care. 2013;17(5):R197 Epub 2013/09/13. 10.1186/cc12891 24025259 PMC4056896

[7] Kruse O, Grunnet N, Barfod C. Blood lactate as a predictor for in-hospital mortality in patients admitted acutely to hospital: a systematic review. Scandinavian journal of trauma, resuscitation and emergency medicine. 2011;19:74 Epub 2011/12/29. 10.1186/1757-7241-19-74 22202128 PMC3292838

[8] Trzeciak S, Dellinger RP, Chansky ME, Arnold RC, Schorr C, Milcarek B, et al Serum lactate as a predictor of mortality in patients with infection. Intensive care medicine. 2007;33(6):970–7. Epub 2007/04/14. 10.1007/s00134-007-0563-9 . 17431582

[9] Gaieski DF, Goyal M. Serum lactate as a predictor of mortality in emergency department patients with infection: does the lactate level tell the whole story? Annals of emergency medicine. 2005;46(6):561–2; author reply 2. Epub 2005/11/26. 10.1016/j.annemergmed.2005.07.021 . 16308078

[10] Arnold RC, Shapiro NI, Jones AE, Schorr C, Pope J, Casner E, et al Multicenter study of early lactate clearance as a determinant of survival in patients with presumed sepsis. Shock. 2009;32(1):35–9. Epub 2009/06/18. . 19533847 10.1097/shk.0b013e3181971d47

[11] Rachoin JS, Weisberg LS, McFadden CB. Treatment of lactic acidosis: appropriate confusion. Journal of hospital medicine. 2010;5(4):E1–7. Epub 2010/04/16. 10.1002/jhm.600 . 20394011

[12] Kim HJ, Son YK, An WS. Effect of sodium bicarbonate administration on mortality in patients with lactic acidosis: a retrospective analysis. PloS one. 2013;8(6):e65283 Epub 2013/06/12. 10.1371/journal.pone.0065283 23755210 PMC3673920

[13] Bone RC, Balk RA, Cerra FB, Dellinger RP, Fein AM, Knaus WA, et al Definitions for sepsis and organ failure and guidelines for the use of innovative therapies in sepsis. The ACCP/SCCM Consensus Conference Committee. American College of Chest Physicians/Society of Critical Care Medicine. Chest. 1992;101(6):1644–55. Epub 1992/06/01. . 1303622 10.1378/chest.101.6.1644

[14] Ferreira FL, Bota DP, Bross A, Melot C, Vincent JL. Serial evaluation of the SOFA score to predict outcome in critically ill patients. Jama. 2001;286(14):1754–8. Epub 2001/10/12. . 11594901 10.1001/jama.286.14.1754

[15] Knaus WA, Draper EA, Wagner DP, Zimmerman JE. APACHE II: a severity of disease classification system. Critical care medicine. 1985;13(10):818–29. Epub 1985/10/01. . 3928249

[16] Fluss R, Faraggi D, Reiser B. Estimation of the Youden Index and its associated cutoff point. Biometrical journal Biometrische Zeitschrift. 2005;47(4):458–72. Epub 2005/09/16. . 16161804 10.1002/bimj.200410135

[17] Zeger SL, Liang KY. Longitudinal data analysis for discrete and continuous outcomes. Biometrics. 1986;42(1):121–30. Epub 1986/03/01. . 3719049

[18] De Backer D. Lactic acidosis. Intensive care medicine. 2003;29(5):699–702. Epub 2003/04/19. 10.1007/s00134-003-1746-7 . 12682722

[19] Madias NE. Lactic acidosis. Kidney international. 1986;29(3):752–74. Epub 1986/03/01. . 3702227 10.1038/ki.1986.62

[20] Ince C. The microcirculation is the motor of sepsis. Crit Care. 2005;9 Suppl 4:S13–9. Epub 2005/09/20. 10.1186/cc3753 16168069 PMC3226164

[21] Levy B, Desebbe O, Montemont C, Gibot S. Increased aerobic glycolysis through beta2 stimulation is a common mechanism involved in lactate formation during shock states. Shock. 2008;30(4):417–21. Epub 2008/03/08. 10.1097/SHK.0b013e318167378f . 18323749

[22] Levraut J, Ciebiera JP, Chave S, Rabary O, Jambou P, Carles M, et al Mild hyperlactatemia in stable septic patients is due to impaired lactate clearance rather than overproduction. American journal of respiratory and critical care medicine. 1998;157(4 Pt 1):1021–6. Epub 1998/05/01. 10.1164/ajrccm.157.4.9705037 . 9563714

[23] Mikkelsen ME, Miltiades AN, Gaieski DF, Goyal M, Fuchs BD, Shah CV, et al Serum lactate is associated with mortality in severe sepsis independent of organ failure and shock. Critical care medicine. 2009;37(5):1670–7. Epub 2009/03/28. 10.1097/CCM.0b013e31819fcf68 . 19325467

[24] Susantitaphong P, Sewaralthahab K, Balk EM, Jaber BL, Madias NE. Short- and long-term effects of alkali therapy in chronic kidney disease: a systematic review. American journal of nephrology. 2012;35(6):540–7. Epub 2012/06/02. doi: 10.1159/000339329 000339329 22653322 10.1159/000339329 PMC3580168

[25] Valenza F, Pizzocri M, Salice V, Chevallard G, Fossali T, Coppola S, et al Sodium bicarbonate treatment during transient or sustained lactic acidemia in normoxic and normotensive rats. PloS one. 2012;7(9):e46035 Epub 2012/10/03. 10.1371/journal.pone.0046035 23029373 PMC3461035

[26] Dellinger RP, Levy MM, Rhodes A, Annane D, Gerlach H, Opal SM, et al Surviving sepsis campaign: international guidelines for management of severe sepsis and septic shock: 2012. Critical care medicine. 2013;41(2):580–637. Epub 2013/01/29. 10.1097/CCM.0b013e31827e83af . 23353941

[27] Jansen TC, van Bommel J, Schoonderbeek FJ, Sleeswijk Visser SJ, van der Klooster JM, Lima AP, et al Early lactate-guided therapy in intensive care unit patients: a multicenter, open-label, randomized controlled trial. American journal of respiratory and critical care medicine. 2010;182(6):752–61. Epub 2010/05/14. 10.1164/rccm.200912-1918OC . 20463176

[28] Luft FC. Lactic acidosis update for critical care clinicians. Journal of the American Society of Nephrology: JASN. 2001;12 Suppl 17:S15–9. Epub 2001/03/17. . 11251027

[29] Garcia-Alvarez M, Marik P, Bellomo R. Sepsis-associated hyperlactatemia. Crit Care. 2014;18(5):503 Epub 2014/11/15. 10.1186/s13054-014-0503-3 25394679 PMC4421917

